# Genome Sequence of *Vaccinia virus* Strain Lister-Butantan, a Lister Vaccine Variant Used during a Smallpox Eradication Campaign in Brazil

**DOI:** 10.1128/genomeA.00536-16

**Published:** 2016-06-23

**Authors:** Felipe Assis, Giliane Trindade, Betânia Drumond, Mike Frace, Scott Sammons, Ginny Emerson, Yu Li, Darin Carroll, Dhwani Batra, Jonatas Abrahão, Erna Kroon

**Affiliations:** a Laboratório de Vírus do Instituto de Ciências Biológicas da Universidade Federal de Minas Gerais, Belo Horizonte, Minas Gerais, Brazil; b National Center for Emerging and Zoonotic Infectious Diseases (NCEZID), Centers for Disease Control and Prevention, Atlanta, Georgia, USA

## Abstract

Here, we report the 187.8-kb genome sequence of *Vaccinia virus* Lister-Butantan, which was used in Brazil during the WHO smallpox eradication campaign. Its genome showed an average similarity of 98.18% with the original Lister isolate, highlighting the low divergence among related *Vaccinia virus* vaccine strains, even after several passages in animals and cell culture.

## GENOME ANNOUNCEMENT

Among strains used in the Smallpox Eradication Programme, Lister-derived strains were by far the most widely distributed around the world (1). Due to this, several different strains were generated in different countries, leading ultimately to a slightly divergent variants of the Lister Elstree original strain (LO) (2, 3). In Brazil, some laboratories received financial support for the development of freeze-dried vaccine production during earlier years of the national smallpox eradication campaign (1, 4). In this work, we report the genome sequence of vaccine *Vaccinia virus* strain Lister-Butantan (LBT), a strain largely used during the national smallpox eradication campaign in Brazil.

The LBT genome was extracted using a BioRobot EZ1 workstation and the protocol to purify genomic DNA from tissue (Qiagen, Hilden, Germany). The DNA was used as a template for producing 20 overlapping PCR amplicons. The templates were sequenced by primer walking on both strands using Applied Biosystems BigDye 3.1 chemistry and ABI 3730XL automated DNA (Applied Biosystems, Coralville, IA). The resulting reads covered the genome in a 9-fold average redundancy at each base position. Chromatogram data were assembled using SeqMerge (Accelrys, Inc., Madison, WI), Phred/Phrap was used for base-calling and assembly software, and Consed was used for sequence editing. The FgenesV tool was used for gene prediction (http://www.softberry.com/). The functional annotations were performed using the Blast2GO platform (5). The genome annotation was manually revised. Predicted open reading frames (ORFs) <40 amino acids and with no hits in any database were ruled out.

The LBT genome is a linear double-stranded DNA (dsDNA) molecule of 187,893 bp long, with a G+C content of 33.66%. This molecule was predicted to contain 222 ORFs, with size ranging from 40 to 1,286 amino acids (aa) (average length, 783 aa). The LBT and LO strains showed a mean amino acid identity of 99.47%. Moreover, we identified a total of 535 nucleotide substitutions, of which 206 (38.5%) were nonsynonymous.

Phylogenetic analysis based on the central conserved region of the genome (81 kbp) revealed that LBT is related to *Vaccinia virus* (VV) strain Lister, commonly used in Europe during the smallpox eradication program (6).

Regarding main differences between the LBT and LO strains, we highlight the single nucleotide polymorphisms (SNPs) and/or other kinds of polymorphisms in genes involved in: (i) evasion of host defense mechanisms or virulence: ORF 013 (serine protease inhibitor [SPI-1]), ORF 019 (interleukin 18 [IL-18] binding protein), M2L and K1L genes (NF-kB inhibitor protein), and B2R and B3R genes (“Schaflen genes” family); (ii) structural and enzyme proteins: ORF 103 (thymidine kinase), ORF 164 (ATPase/DNA packaging protein), ORF 182 (thymidylate kinase), ORF 189 (hemagglutinin protein), and ORF 033 (complement control protein—B5R in VACV-Cop); and (iii) proteins recognized by the immune system: ORF 099 (internal virion protein) and ORF 192 (serine-threonine kinase). Despite these polymorphisms, we conclude that these findings corroborate previous studies that showed a close relationship between the Lister-derived strains in terms of DNA and protein sequences, regardless of numerous historical passages and maintenance of vaccine stocks.

### Nucleotide sequence accession number.

The genome sequence of VV strain Lister-Butantan was submitted to GenBank with the accession no. KX061501.
